# Optimizing tissue-clearing conditions based on analysis of the critical factors affecting tissue-clearing procedures

**DOI:** 10.1038/s41598-018-31153-7

**Published:** 2018-08-24

**Authors:** June Hoan Kim, Min Jee Jang, Jungyoon Choi, Eunsoo Lee, Kyung–Deok Song, Jaeho Cho, Keun-Tae Kim, Hyuk-Jin Cha, Woong Sun

**Affiliations:** 1 Department of Anatomy, Korea University College of Medicine, Seoul, 02841 Republic of Korea; 2 Department of Physics, Korea University, Seoul, 02841 Republic of Korea; 3 Center for Molecular Spectroscopy and Dynamics, Institute for Basic Science (IBS), Seoul, 02841 Republic of Korea; 4 Department of Radiation Oncology, Yonsei University College of Medicine, 50 Yonsei-ro, Seodaemun-gu, Seoul, 120-752 Republic of Korea; 5 Department of Life Science, Sogang University, 35th Baekbum-ro Mapo-gu, Seoul, 04107 Republic of Korea; 6 College of Pharmacy, Department of Pharmacy, Seoul National University, Gwanak-ro, Gwanak-gu, Seoul, 08826 Republic of Korea

## Abstract

Tissue-clearing techniques have received great attention for volume imaging and for the potential to be applied in optical diagnosis. In principle, tissue clearing is achieved by reducing light scattering through a combination of lipid removal, size change, and matching of the refractive index (RI) between the imaging solution and the tissue. However, the contributions of these major factors in tissue clearing have not been systematically evaluated yet. In this study, we experimentally measured and mathematically calculated the contribution of these factors to the clearing of four organs (brain, liver, kidney, and lung). We found that these factors differentially influence the maximal clearing efficacy of tissues and the diffusivity of materials inside the tissue. We propose that these physical properties of organs can be utilized for the quality control (Q/C) process during tissue clearing, as well as for the monitoring of the pathological changes of tissues.

## Introduction

Understanding the architecture of an organ or the body as a whole, using cellular resolution, is one of the ultimate goals in biology. The recent explosion of tissue-clearing techniques, which have been successfully applied to glimpse whole or parts of body structures, reflects this demand^1–4^. The enhancement of tissue transparency is achieved by the reduction of light scattering. Theoretically, light scattering occurs due to the inhomogeneity of materials with different refractory indices (RI)^5^ and spatial arrangement of scatters^6,7^. Therefore, tissue clearing requires the reduction of light scattering either by diluting/removing materials that induce light scattering (such as membrane lipids), swelling or shrinkage of tissues to alter the structure of scattering sources such as extracellular matrix (ECM), or by reducing the RI difference, using adjusting media with RI value similar to the average RI of tissue components. All the currently available protocols are designed based on these principles^8^. Considering that organs/tissues have different macromolecule contents and unique histological signatures^9,10^, their optical properties and responses to different tissue-clearing methods should also be different. However, the clearing-related properties of organs have not yet been comprehensively explored, which makes choosing the best clearing methods for the desired organs difficult.

To this end, we developed assay systems to analyze the differential clearing properties of organs/tissues and to evaluate the quality of tissue clearing based on the changes in their macromolecule components and transparency. This battery of assays allows for the systematic monitoring of the clearing processes, which can then be used as quality control methods for the standardization of the clearing process. Furthermore, our protocols can be used to verify the pathological changes of tissues, which will be a foundation for the label-free diagnosis of tissues based on optical clearing methods.

## Results

### Differential contribution of factors affecting tissue clearing

**Figure 1:**
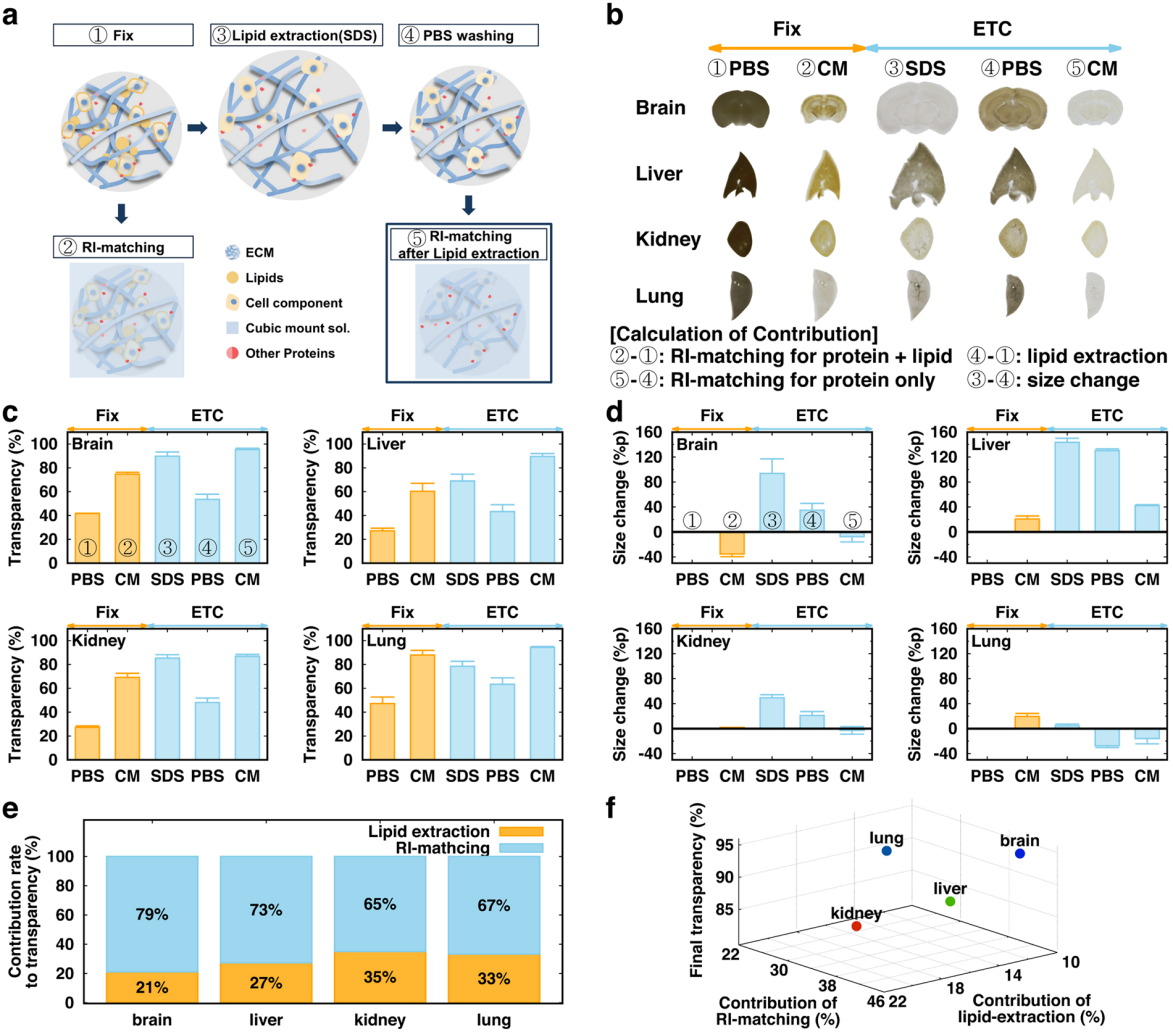
ACT process and contributions of lipid-extraction, RI-matching, and size expansion to tissue transparency **(a)** Schematic diagram of refractive index (RI) matching and the active CLARITY technique (ACT) process. **(b)** Images of 1-mm-thick tissue slices during the tissue-clearing process. Comparison of two different steps provides contributions of each factor (bottom) **(c)** Transparency of each organ tissue during the tissue-clearing process. **(d)** Size changes of each organ at each tissue-clearing step. **(e)** Contributions of RI-matching and lipid extraction to tissue clearing, if the effect of size change on transparency is compensated. **(f)** 3D plot of contributions of lipid-extraction and RI-matching to tissue transparency. The color indicates the final transparency (red-green-blue). N = 3 for each organ.

To evaluate the contributions of major factors, we employed our recently published active clarity technique (ACT) for tissue clearing^4^. We re-defined the ACT process into 5 steps as shown in Fig. 1a,b: 1) fixation and polymerization, 2) RI-matching before lipid-extraction, 3) electrophoretic tissue clearing (ETC) in Sodium dodecyl sulfate (SDS) solution to remove lipid from the tissue, 4) washing with phosphate buffer saline (PBS) after ETC, and 5) RI-matching after ETC. Changes in 1-mm-thick tissues from the brain, liver, kidney, and lung in each step are shown in Fig. 1b. The transparency and size of four tissues in each step were quantified (Fig. 1c,d). The thickness of the specimens exhibited strong negative correlation with their transparency under the same RI-matching condition (Suppl. Fig. 1), and we chose 1-mm thickness for further analyses. Based on these measurements, we calculated the contributions to tissue transparency of: 1) lipid extraction, by comparing the transparency of tissues in PBS before ETC (①) with that of tissues washed by PBS after the ETC step (④); 2) RI-matching, by comparing the transparency of a tissue in PBS (④) with that in CUBIC-mount solution (CM)^4^ (⑤); and 3) size expansion, by comparing the transparency of a tissue in SDS after ETC (③) with that of a tissue in PBS (④). The difference in transparency obtained by the last comparison was primarily owing to the tissue expansion, because the RIs of SDS solution and PBS were similar (1.3339 vs. 1.3343). We additionally compared the tissue size and transparency in various concentrations of PBS (0.1–10×) and found that the correlation between size change and transparency was very similar in all organs (Suppl. Fig. 2). However, the cleared tissue in CUBIC-mount solution returned to its original size, and we obtained the contributions of lipid extraction and RI-matching to tissue transparency for each organ by normalizing the scores based on their tissue size at the given step (Fig. 1e). The relationships of the contributions of lipid extraction and RI matching to the final transparency of different organs are shown in Fig. 1f, illustrating the differences in the tissue-clearing properties.

In general, the contribution rate of lipid extraction is smaller in organs which exhibited larger swelling in size (brain, liver), comparing to organs exhibiting less change (kidney, lung). In fact, the change of transparency by RI-matching can be achieved before ETC (with lipid; ②-①) and after ETC (after removal of lipids, ⑤-④). The contribution of RI-matching to tissue transparency increased in “large-size-change” tissue (brain, liver) by ETC, but decreased in “small-size-change” tissue (kidney, lung) (Suppl. Fig. 3). Thus, we reasoned that lipid extraction made tissue more porous and expandable, and RI solution will easily infuse and replace the void volume of an ETC-processed tissue. On the other hand, the RI-matching effect should decrease after lipid extraction, because of the relatively high RI of lipids (RI 1.44–1.49;^11^). Thus, this trade-off in lipid extraction effects seems to differentially influence the tissue-clearing process.

### Evaluation of lipid extraction

**Figure 2:**
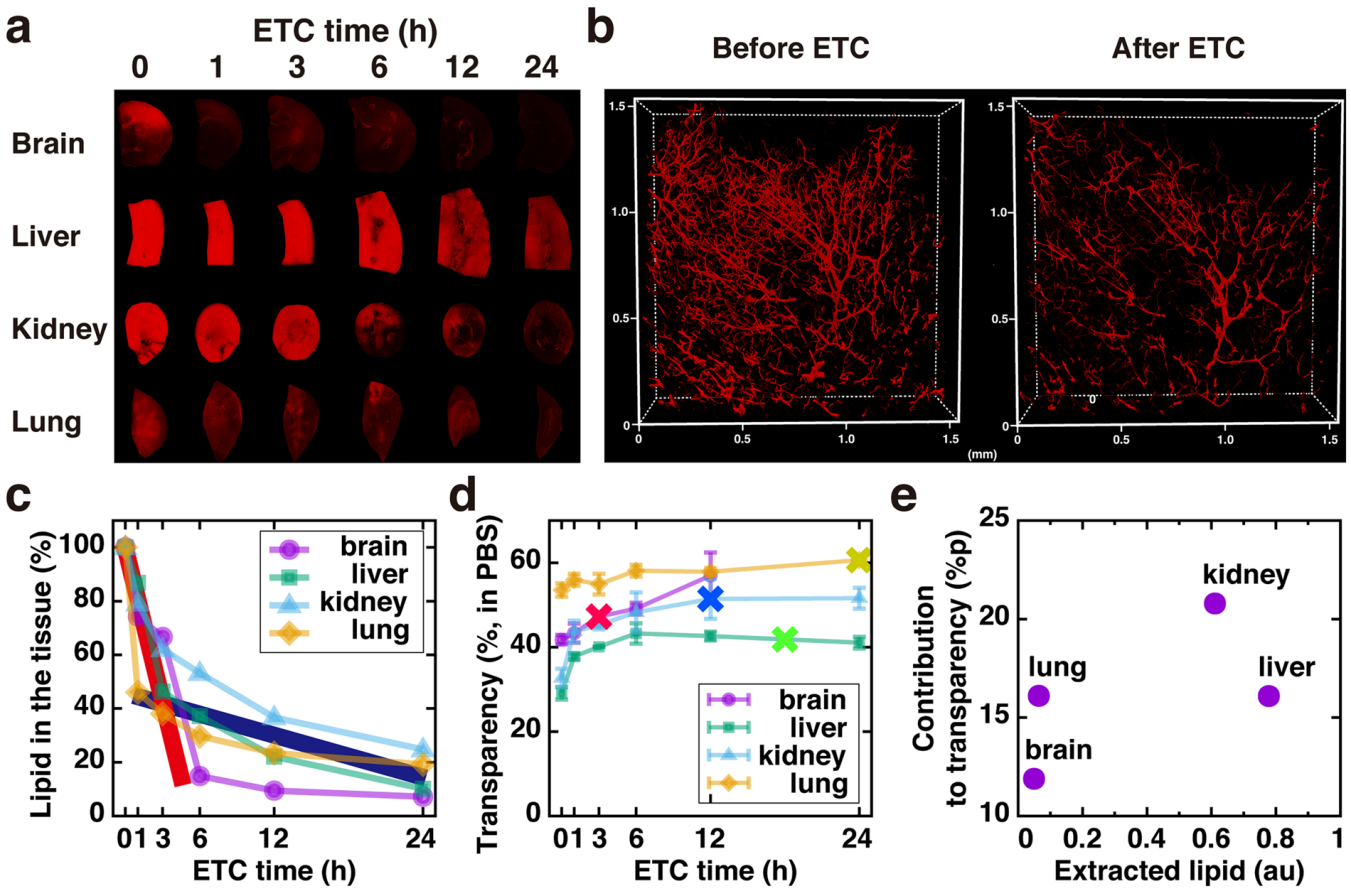
Evaluation of lipid extraction **(a)** Images of DiI-stained tissue at different ETC time points. **(b)** Pre-labeled CM-DiI signals remaining in the brain vasculature before (left) and after 2 hr ETC (right). Lipid extraction rate **(c)** and transparency **(d)** at different ETC time points. “X” marked points in **(d)** indicate an optimal time point for each organ for maximal clearing. N = 3, 3, 6, 6 for brain, lung, liver and kidney, respectively. **(e)** Correlation graph of extracted lipid and contribution of lipid extraction to the improvement of the transparency of each organ.

As a step toward understanding the precise mechanism by which the lipid extraction step differentially contributes to tissue clearing, we first investigated the changes in transparency during ETC. DiI [(2*Z*)-2-[(*E*)-3-(3,3-dimethyl-1-octadecylindol-1-ium-2-yl)prop-2-enylidene]-3,3-dimethyl-1-octadecylindole; perchlorate] is commonly used for visualizing lipids in the cell membrane. We established an assay measuring the level of DiI-labeled lipids in each specimen and found that it was well correlated with the amount of cells estimated by DNA contents in the tissues (Suppl. Fig. 4), suggesting that the DiI level faithfully represents the amount of lipids associated with cell membranes. Fig. 2a,c show that the extraction profiles of DiI-labeled lipids during ETC are different in each organ. Interestingly, two different extraction rates were observed in all organs. Fast extraction occurred for 1–3 hr of ETC (thick red line in Fig. 2c), and thereafter, slow extraction was seen (thick blue line in Fig. 2c), suggesting that a subset of DiI-labeled lipids are efficiently extracted by ETC with SDS buffer but some other species of lipids are extracted much more slowly. Accordingly, when CM-DiI was pre-loaded to the vasculature of a mouse brain by cardiac perfusion before ETC, substantial DiI signals were remained after 2 hr of ETC when slow extraction was not yet completed (Fig. 2b). Furthermore, the extraction profile of other lipid species labeled with Oil-Red-O (ORO) was markedly different from that of DiI-labeled lipids (Suppl. Fig. 5). Therefore, the transparency of four organs at different ETC time points was not well correlated with the extraction rate of either DiI-labeled or ORO-labeled lipids (Fig. 2d; Suppl. Fig. 5), and the total amount of extracted DiI-labeled lipids exhibited no correlation with the contribution of lipid extraction to the transparency change in each organ (Fig. 2e). Collectively, ETC-based lipid extraction can be achieved by fast extraction in 1–6 hr for most organs, and an additional 12 hr of ETC might be required for some organs in which lipid extraction greatly contributes to the tissue clearing.

### Evaluation of tissue ECM components and RI matching

**Figure 3:**
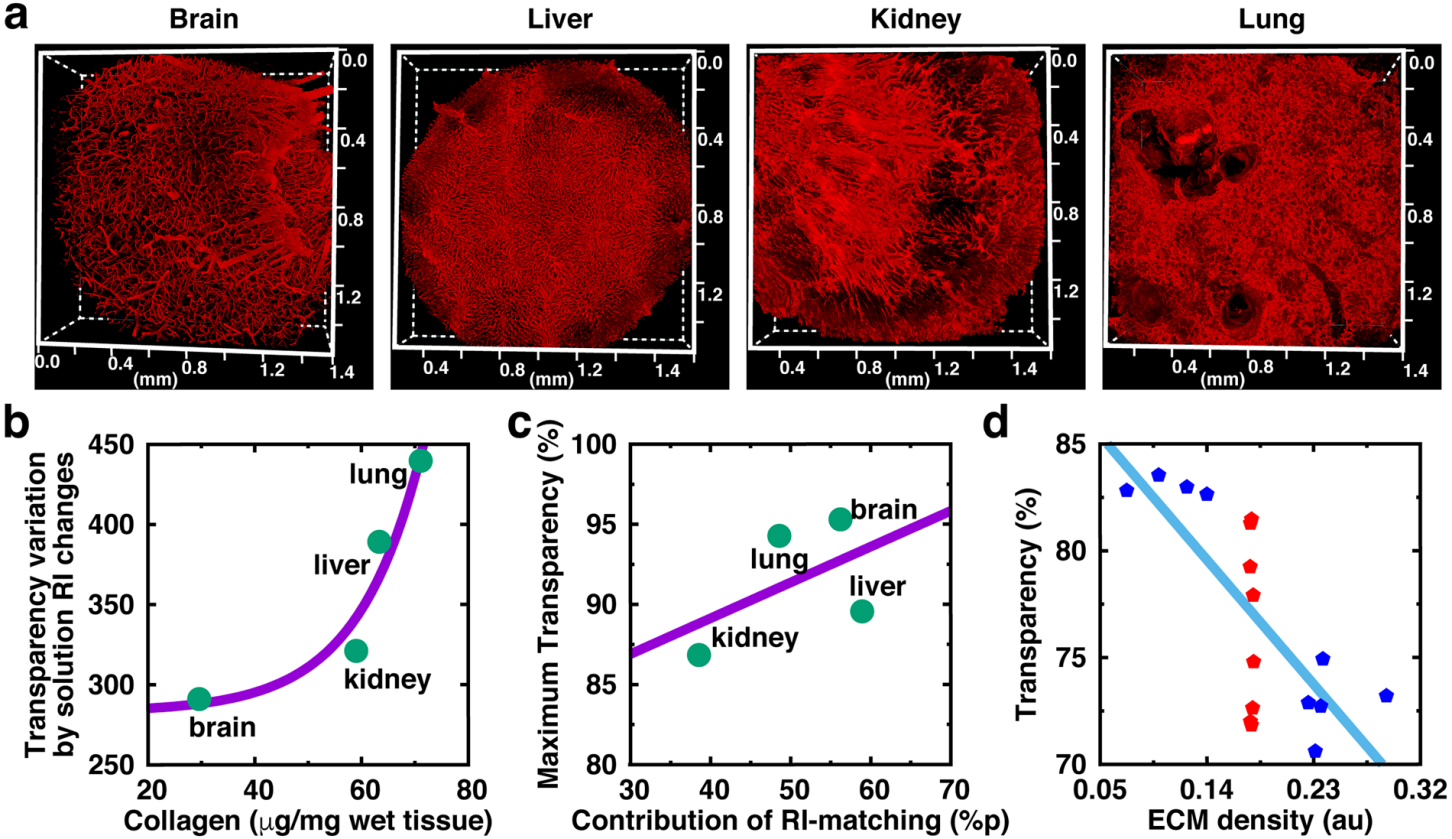
Evaluation of collagen IV and RI matching **(a)** Immunostained images of each organ with collagen IV, which is one of the main components for light scattering in tissues. **(b)** Correlation between the actual collagen amount and variation of transparency on different levels of RI-matching. Also see Suppl. Fig. 6. Average values of triplicated samples were used. **(c)** Maximum transparency of fully cleared tissue determined by contribution of RI-matching (ECM). **(d)** Finite-difference time-domain (FDTD) simulation with randomly generated images of various ECM density showing that light transmittance is dependent on ECM density (from negative slop of the linear fitting line) and ECM structures (from red points having similar ECM density).

The major source of light scattering in lipid-removed tissues may be extracellular matrix proteins (ECM), which are the richest tissue components in most organs. Thus, we explored the relationship between ECM components and RI matching in different tissues. Immuno-labeling images of collagen IV provide an overview of the differences in the density and structures of the ECM in different organs (Fig. 3a). The changes of tissue transparency in solutions with different RI scores were measured and their correlation slope was obtained (Suppl. Fig. 6). The transparency variation based on solution RI changes was highly correlated with the amount of collagen IV in specimens (Fig. 3b), indicating that ECM components are the main determinants of RI matching-based tissue clearing. Interestingly, the contribution of RI-matching calculated in Fig. 1 fitted with the maximum transparency of fully cleared tissues (Fig. 3c), but the orders of tissues in Fig. 3b (brain < kidney < liver < lung) and Fig. 3c (kidney < lung < brain < liver) are different. Thus, we speculated that not only the total amount but also the arrangements of the ECM contribute to RI matching-based tissue clearing. Because ECM architecture cannot be experimentally modified, a finite-difference time-domain (FDTD) simulation with randomly generated images (Suppl. Fig. 7) for various ECM densities was designed to address this issue. As a result, both the density and structure of the ECM were considered to have major roles in light transmittance (transparency). Light transmittance showed a linear correlation to ECM density with negative slop, whereas quite a wide range of light transmittance was shown in similar-density ECM (Fig. 3d).

### Establishment of a molecular diffusion-based evaluation method for the ACT-processed specimen

**Figure 4:**
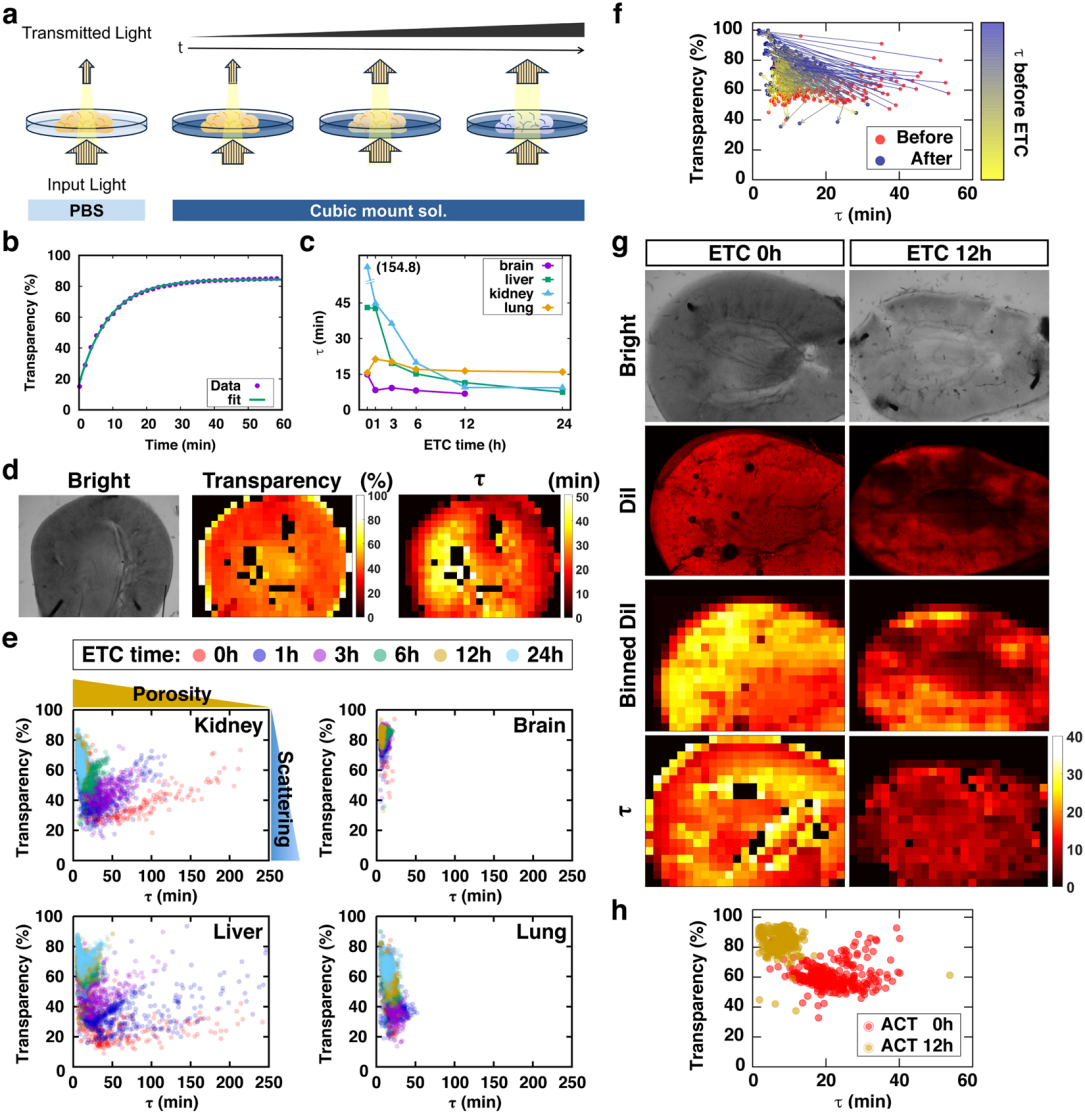
Molecular diffusion-based evaluation method for tissue clearing **(a)** Conceptual image showing the evaluation process of tissue clearing with two variables: transparency and time constant (τ) for the diffusion of RI-matching solution. **(b)** Time-transparency data fitted to one-phase association function to get time constant and final transparency. **(c)** τ profile of each organ at different ETC time points. **(d)** Sample images of the same mouse kidney slice: bright-field image (left), transparency map (middle), τ map (right). **(e)** τ-transparency scatter plot, which could indicate the optical properties of a cleared tissue. Each data points were obtained from 3 independent samples in each organ at each ETC time point. **(f)** τ and transparency relations for each points of a kidney sample before (red dots) and after 12 hr ETC (blue dots) and their position change (colored arrows). The colors of arrow were from τ values before ETC. **(g)** Images of mouse kidney at ETC 0 and 12 hr. **(h)** Scatter plot of τ-transparency from samples in **(g)**.

As mentioned above, specimens with the same transparency would have different conditions (e.g., different level of lipid removal). Cleared specimens are often subject to further processing for optical examinations with fluorescent labeling of specific macromolecules^1,3,4,12–14^. Because these labeling methods include the diffusion of probes/dyes into the tissues, it is especially important to verify the porosity of the cleared tissue samples. However, the tissue transparency is determined by multiple factors, and it does not indicate the quality of the tissue. Thus, we propose a simple method to evaluate the status of tissues by measuring their transparency and the diffusion rate of RI-matching solution which are composed of micro-molecules, such as glucose and ethylene glycol whose diffusion properties were well documented by the recent study^15^. The experimental process is summarized in Fig. 4a. First, a specimen in PBS after ETC is placed in an imaging setup, such as a microscope, or a simple system in a dark room composed of a backlight and camera, which collects the transmitted light. PBS solution is then replaced by RI-matching solution, and time-lapse images are acquired until tissue clearing reaches the plateau. The time-lapse images are segmented into many square tiles with a fixed unit size (i.e., 50 μm x 50 μm). The changes in local transparency of each tile are then automatically calculated to deduce the maximum transparency and time constant (τ) by fitting the time series to the one-phase association function (Fig. 4b). The estimated τ values in all tissues progressively decreased as ETC duration increased, suggesting that the τ values are proportional to the tissue porosity, which is increased by the removal of lipids (Fig. 4c). The comparisons of transparency and τ maps (middle and right in Fig. 4d, respectively) showed gross similarity, but they were not completely matching owing to the local concentration difference of ECM/proteins, which were not extracted by ETC. Thus, the τ-transparency scatter plot of each tile clearing demonstrates the changes/status of the specimens (Fig. 4e). In this plot, the dots are spotted from top to bottom depending on the amount of light scattering by the tissue area (tile), whereas the dots are positioned right to left as the degree of porosity increases. Indeed, a shift of the spots from the lower-right to upper-left side was observed in tissues with the progression of ETC (Fig. 4f). To confirm the relationship between τ (porosity) and lipid extraction, the binned map of DiI fluorescent image was compared with the τ map of the sample tissue (Fig. 4h), showing same shift of spots from lower-right to upper-left (Fig. 4g). As a result, correlation coefficients between the maps of DiI and τ were 0.71 (0 hr) and 0.59 (12 hr), indicating that τ mainly reflects the distribution of lipid in a tissue. Therefore, the profile of τ-transparency plots can be used for evaluating the degree of lipid extraction from a tissue.

### Application of tissue-clearing procedure for the diagnosis of tissue injury

**Figure 5:**
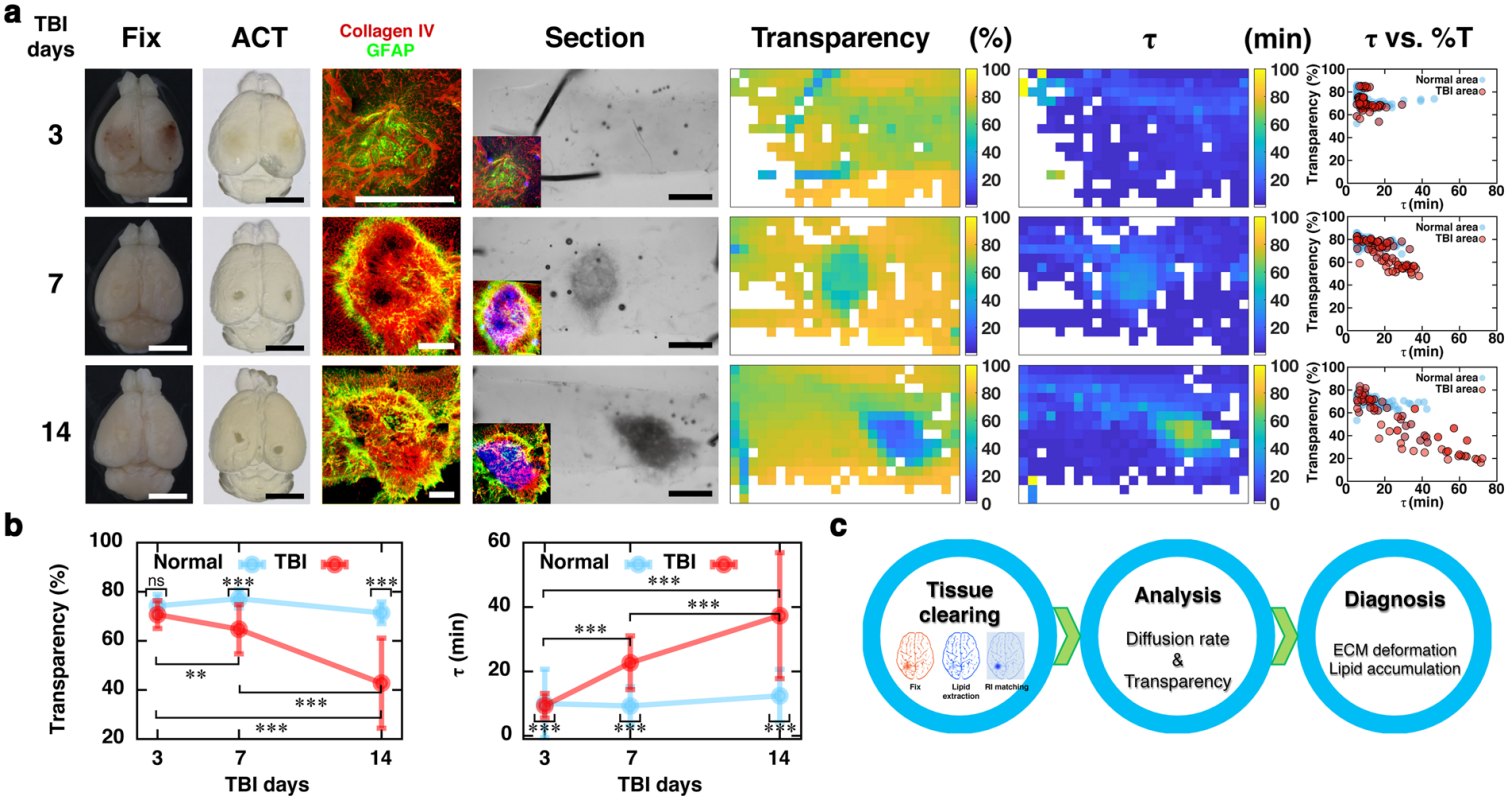
Diagnostic application of tissue clearing **(a)** Images of mouse brain 3, 7, and 14 days after cryogenic TBI. (From left to right) bright-field images of whole brain before (Fix) and after tissue clearing (ACT); section images of injured brain regions stained with collagen IV (red; ECM accumulation), GFAP (green; reactive astrogliosis); 1-mm-thick brain slice including both injured and normal regions; transparency and τ maps; and scatter plot of τ-transparency showing the optical condition of the tissues. Insets show the superimposed images of immunolabelings and optical contrast (pseudocolored with inversed blue). The sizes of scale bars in **(a)** are 5 mm for Fix and ACT, 400 μm for immunostaining, and 1 mm for Section. **(b)** Transparency and τ profiles of normal and injured areas at 3, 7, and 14 days after TBI. Mean ± SD for tiles of normal area at TBI days 3, 7, 14 (N = 37, 71, 68 respectively) and injury area at TBI days 3, 7, 14 (N = 89, 54, 46 respectively). **P < 0.01, ***P < 0.001 compared to control determined by one-way ANOVA with post-hoc Tukey **(c)** Proposed diagnostic process for deformed tissue, using tissue clearing and τ measurement.

Finally, we tested whether changes in the optical status of tissues during the clearing procedure could be used to evaluate tissue quality under pathological conditions. Traumatic brain injury (TBI) is known to cause astrogliosis and tissue remodeling, including the deposit of ECM molecules at the penumbra area of the injury spots^16–18^. Accordingly, tissue clearing enhanced the contrast between the normal vs. injured area (Fig. 5a). Similarly, enhanced optical contrast with fibrosis was also found in the focal lung fibrosis model (Suppl. Fig. 8). This enhanced optical contrast coincides with the deposit of collagens in the injured area (insets, Fig. 5a), whereas the time-course for cell accumulation and gliosis appears to be earlier than that for the development of optical contrasts^19^. In the sectioned tissues, the τ-transparency map demonstrated that the injured area moved toward the lower-right position (Fig. 5a), and the average transparency and τ values were accordingly reduced and increased, respectively (Fig. 5b). These results indicate that ECM deposition occurred in the injured area, which can be captured by the currently developed procedure (Fig. 5c).

## Discussion

In this study, we comprehensively analyzed the factors affecting tissue clearing, and we propose novel methods to evaluate the status of tissue clearing, which also can be used as markers for tissue injury. Optical tissue clearing is achieved by the combination of 3 major factors: lipid removal, size expansion, and RI adjustment. Because the composition and structure of tissues are different, the contributions of these factors on tissue clearing are also tissue-specific.

Among the sample organs we tested, the contribution of lipid extraction was high in the kidney and lung, whereas low contribution was found in the liver and brain. Thus, satisfactory optical clearing can be achieved by simple RI adjustment in some tissues for which the lipid contribution is low. Accordingly, as a study reported that simple immersing a tissue into saline was sufficient to achieve local RI matching for lung tissue^20^, several tissue-clearing techniques such as SeeDB^21^ and Scale^22^, which do not include a lipid extraction step, have been successfully used for brain tissue clearing. In contrast, organs in which lipid extraction has a higher contribution should be processed using techniques that include lipid extractions, such as CLARITY^1^, iDISCO^2^, and CUBIC^3^. We also found that the kinetics of lipid extraction is not linear, and at least two different extraction kinetics of DiI-labeled lipid components were recognized, although the exact identification of these molecules needs to be further explored. We reasoned that there are roughly two different groups of DiI-labeled lipids: SDS-extractable (fast component) or SDS-insoluble (slow component). Removal of the fast component could be achieved within 1–3 hr in all the organs we tested, whereas the remaining components were slowly removed in >24 hr. Thus, it is difficult to remove these slow components of lipids completely using SDS-based ETC methods, and extended ETC (>24 hr) might cause tissue damage. Therefore, these would serve as a limiting factor for thicker tissue clearing using the ACT protocol. It should also be noted that some lipid components cannot be efficiently recognized by DiI. For instance, lipid droplets in the cells were not labeled with DiI, and lipids in adipose tissues were not efficiently removed by SDS (Suppl. Fig. 5). Therefore, the complete removal of lipid species is not achievable when using an SDS-based protocol, and organic solvent-based methods such as DISCO series^2,23,24^ or CUBIC^3^ should be considered. On the other hand, the remaining lipophilic CM-DiI in the ACT protocol can be used for the post-clearing detection of DiI signals such as vasculatures as shown in Fig. 2b. However, because extended ETC will progressively remove DiI signal from weakly labeled capillaries, caution is necessary for using this technique for high-fidelity, quantifiable analysis.

Size changes during tissue clearing vary depending on the technique used. Because tissue expansion results in the dilution of materials causing light scattering, hyper-hydration of tissues in urea solution has been utilized to enhance the transparency of tissues. ETC also causes mild tissue expansion, which transiently affected tissue transparency. With this advantage, expansion microscopy was introduced^25^. However, a size change is not desirable in many applications because of the distortion caused by anisotropic expansion/shrinkage, and there are modified versions of tissue clearing with adjustments made to the clearing solution to prevent size changes. Interestingly, we found that different organs exhibit unique swelling property, which do not appear to be associated with tissue softness or ECM contents. For example, lung is relatively soft-tissue organ, but size expansion was relatively small. We speculate that sponge-like structure of lung somehow prevents external expansion from hyper-hydration, and the tissue organization appear to be an important factor for tissue swelling. In the ACT procedure, the cubic-mount solution is also adjusted to maintain the original tissue size, and the contribution of tissue expansion is negligible. In view of the size, it is interesting that the use of organic solvents in iDISCO induces marked tissue shrinkage with enhanced tissue transparency^2,6,26^. It appears that organic solvents causes strong dehydration, and shrinkage owing to osmotic pressure by dehydration^15^ induces quasi-crystallization of the remaining tissue components. Thus, the homogeneity of the component should increase, and the light scattering should be reduced. Based on this notion, recently we demonstrated that complete drying of ACT-processed samples can transform them into film-like, highly transparent thin sheet, which can be used to reduce imaging burden^26^.

After lipid removal, the RI of ACT-processed tissues are primarily determined by the protein component in tissues. Since ECM proteins are the most abundant species of proteins in organs, it appears that the amount of ECM proteins is highly correlated with the contribution of RI matching. However, as discussed above, the homogeneity of the tissue (i.e., arrangements of the ECM in organs) should be a factor influencing tissue transparency, and the mathematical simulation supported this notion. Because ECM arrangements are unique signatures of each organ, these results indicate that organs have unique features of tissue clearing based on their protein composition, ECM contents, and ECM arrangements. Both lipid extraction and ECM arrangements are important factors affecting the diffusion of materials^27^. In fact, the homogeneous diffusion of antibody deep into a thick tissue or whole organ is crucial for imaging the specific target labeled by a fluorescence protein. For this reason, measuring tissue porosity is important, but an appropriate method is currently lacking. In this respect, the analysis of τ-transparency relationships, including spatial information, provides a simple and reliable parameter to evaluate tissue porosity and the degree of tissue clearing. Additionally, analyzing the slop of scattered points from lower-left to upper-right in τ-transparency plot of an uncleared tissue can give hints about the ETC time to be needed. It is important to note that the diffusion kinetics of the optical clearing reagents are inversely associated with the kinetics of water flux from the specimen^15,28^. Accordingly, de-lipidation causes tissue hydration and swelling, while optical clearing reagent reverses it via water efflux. For more accurate prediction, regular shaping of specimens or de-coloration step^29^ for pigmented organ tissues such as liver and kidney might be helpful.

The measuring light scattering and diffusion of optical clearing solution for *ex vivo* diagnosis had been proposed^28,30^. Likewise, changes in tissue architecture due to the pathological deposition of ECM can be efficiently monitored by simple tissue clearing and the measurement of transparency. The diagnostic application of tissue-clearing methods has been proposed in many studies, because 3D imaging provides precise volume information of intact tissue^13,14,31^, compared with the conventional sectioning methods. Most proposed methods that utilize tissue clearing for diagnosis are based on fluorescence imaging with enhancement of transparency^32–34^. On the other hand, we propose that optical properties after tissue clearing *per se* can be used for the label-free assessment of tissue conditions and an optical diagnosis. In addition to our current demonstrations in TBI and pulmonary fibrosis models, our method can be applied to other diseases exhibiting ECM modifications which include cancer^28^ and dermatopathy^30^.

In summary, multiple factors affecting tissue transparency during the tissue-clearing process of the brain, liver, kidney, and lung were comprehensively investigated, and how the specific characteristics of different organ tissues contribute to the reduction of light scattering or the improvement of tissue porosity was clarified. We propose that this information can be used to develop a novel quality control (Q/C) process for tissue clearing and diagnosing tissue deformation.

## Methods

### Tissue preparation and tissue clearing

All animal husbandry, animal care, and euthanasia protocols were in accordance with guidelines from the Korea University and have been approved by members of the Korea University Institutional Animal Care and Use Committee (KUIACUC-20150520-1). Mouse organs were cleared with ACT as described in a previous study^4^. Briefly, 7-week-old C57BL/6 mice (DAEHAN Biolink, Inc, Korea.) were transcardially perfused with PBS and paraformaldehyde (PFA), and fixed organ samples were dissected and incubated in hydrogel solution overnight at 4 °C. Hydrogel-infused organs were polymerized for 2–3 hr after degassing. Polymerized samples were cut into 1-mm-thick tissue slices and electrophoresed for fast removal of lipid, using an ETC apparatus (X-CLARITY, Logos systems, Republic of Korea) with following conditions: 1.5 mA, 37 °C. Electrophoretic times for maximal lipid extraction from organ tissues without significant tissue damage were 3, 18, 12, and 24 hr for brain, liver, kidney, and lung tissues, respectively. Brain tissue was cut into coronal slices, whereas other organs were sliced transversally. For RI-matching to achieve clearer images, CUBIC-mount solution [250 g sucrose (50%, w/v), 125 g urea (25%, w/v), and 125 g N,N,N′,N′ -tetrakis (2-hydroxypropyl)ethylenediamine (25%, w/v) dissolved in 150 ml of dH_2_O and brought up to 500 ml] was used^4^.

### Measurement of transparency

Fixed adult mouse organs were cut into 1-mm-thick slices. The slices were imaged using a conventional camera before and after clearing. We measured the size and transparency of the cleared 1-mm-thick organ slices, which were outlined and calculated using ImageJ (NIH, USA). The gray value of the cleared sample image was used to measure tissue transparency, and transparency was normalized to the background part of the image. For τ measurements, transparency was acquired by fitting with the one-phase association function [see Methods section Time constant (τ) of diffusion measurement].

### Calculation of contributions of factors to transparency

The contributions of factors to tissue transparency were calculated the difference in transparency between the two different steps. When T_n_ is transparency at the step n (①-⑤), the contribution of the factor α (C_α_) is followed,

1 $${{\rm{C}}}_{{\rm{Lipid}}}={{\rm{T}}}_{4}-{{\rm{T}}}_{1}$$

2 $${{\rm{C}}}_{{\rm{RI}}-{\rm{matching}}}={{\rm{T}}}_{5}-{{\rm{T}}}_{4}$$

3 $${{\rm{C}}}_{{\rm{Size}}}={{\rm{T}}}_{3}-{{\rm{T}}}_{4}$$

### Measurement of size change

To evaluate the effect of size change on transparency of cleared tissues independently without RI-matching, it is crucial that the refractive index of the solutions used for changing size of tissue have to be preserved. For that, we used different concentration of PBS (0.1×, 0.5×, 1×, 5× and 10×) and 4% SDS, whose refractive indices were 1.3328, 1.3331, 1.3343, 1.3397, 1.3468 and 1.3339 respectively. All refractive indices in this study were measured at 589 nm by ATAGO PAL-RI (ATAGO CO., LTD., Tokyo, Japan).

### Measurement of lipid in tissue using DiI staining

DiI (468495, Sigma-Aldrich, USA) is known to bind phospholipid; hence, we used it to measure the amount of phospholipid in a tissue. The tissue was immersed in 100% dimethyl sulfoxide (DMSO) for enough time to improve the homogenous loading DiI deep into the tissue, after which it was again immersed in 0.2 mM DiI overnight. Finally, the tissue was washed in 1% SDS overnight, followed by extraction of lipids by soaking in 100% isopropanol for 4 hr. The extracted fluorescence value was measured using a Micro plate reader (SpectraMax Plus384, Molecular Devices, Sunnyvale, CA, USA) at 550 nm. For confocal imaging, a lower concentration of DiI (0.2 μM) was used to adjust the fluorescence intensity. For labeling of vasculature with lipidophic dye, CM-DiI (Thermo Fisher Scientific, USA) was used, which is aldehyde-fixably modified version of DiI^35^. Adult C57BL/6 mice were anesthetized with urethane and transcardially perfused with 50 ml of 0.9% saline, 20 ml of 0.01% CM-DiI solution followed by 20 ml of 4% paraformaldehyde for fixation. Fixed brains were cut into 1-mm thick coronal slices and were cleared by ACT for 2 hr. Cleared tissue samples were incubated in CUBIC-mouse solution for 1 h. Image was acquired using a TCS SP8 confocal laser-scanning microscope (Leica, Germany) with a 10X lens (NA cq0.3, working distance .32.2 mm) and 568-nm excitation wavelength.

### Measurement of lipid in tissue using Oil-Red-O staining

Oil-Red-O (ORO, O0625, Sigma-Aldrich, USA) is known to bind lipid droplets, so we used it to measure amount of lipid droplets in a tissue. The protocol of ORO staining and extraction was customized for our samples from the previous study^36^. Cleared tissues were immersed in ORO (2 mM in 100% isopropanol, 10 μL/mg) and incubated at room temperature for 2 hours. ORO stained tissues were washed tissue in 1% SDS overnight and lipid was extracted by soaking into 100% isopropanol for 4 hrs, and extracted fluorescence value was measured with Micro plate reader (SpectraMax Plus384, Molecular Devides, Sunnyvale, CA, USA) at 500 nm.

### Immunohistochemistry

Cleared tissue organs were trimmed by 1-mm circular punch and incubated with primary antibody diluted in blocking solution (6% BSA and 0.2% Triton-X100 in 0.1X PBS) for 2 days at 37 °C shaker. Antibodies against Collagen IV (1:500, Millipore) and GFAP (1:1000, DAKO) was used. The samples were washed 3 times with 0.1x PBS, and incubated with secondary antibodies (1:500) for 2 days at 37 °C shaker by matching primary antibody host for fluorescence imaging.

### Collagen quantification

The collagen contents of samples were quantified using a Sircol collagen assay kit (Biocolor, Carrickfergus, UK) according to the manufacturer’s instructions. This step was done following collagen extraction by incubation for overnight in a pepsin-containing solution at 65 °C. The absorbance of each sample was determined at 550 nm using a microplate reader (Molecular Devices, CA, USA) and the collagen quantity was calculated by usage of a standard curve generated with water-soluble denatured collagen from bovine skin.

### Finite difference time domain (FDTD) simulation

To investigate the effect of density and structure of ECM on tissue transparency, finite difference time domain (FDTD) simulation was performed. To describe large and fine virtual ECM structures, random images (45 μm ∗ 90 μm at 30 nm resolution) were created by random walk, following the various scale variable (*γ*) of levy distribution function (characteristic function:

$$E({e}^{itx})=\exp (-{(\gamma |t|)}^{\alpha }[1+i\beta \,\sin (t)\tan \,\frac{\pi \alpha }{2}({(\gamma |t|)}^{1-\alpha }-1)]+i{\delta }_{0}t),\alpha =0.5,\beta =1)$$

by a Matlab code (Suppl. Fig. 7). And for infinite area and finite thickness of virtual ECM structure, a periodic boundary condition was applied along the x-axis and, a perfectly matched layer was applied along y-axis. The refractive indices of background (water) and random dots (ECM) were set to 1.33 and 1.47 respectively. For one optical period, an x-polarized plane wave of 590 nm wavelength was normally incident to a virtual ECM structure, and the light transmittance was calculated by the ratio of transmitted energy to incident energy. For the steady state, the plane waves propagated for 5,000 optical periods. In-house FDTD code was used^37^ for all simulations of light transmittance.

### Time constant (τ) of diffusion measurement

First, the glass bottom of a 35-mm-diameter black confocal dish (102350, SPL, Republic of Korea) was covered with a thin polydimethylsiloxane (PDMS) sheet, onto which a tissue stored in PBS was pinned using a short thin metal wire to prevent it from floating or moving when the solution was exchanged. PDMS is suitable for sample fixing for imaging because of its optical clarity and ease of treatment. The dish was mounted onto the stage of a microscope (EVOS FL, Thermo Fisher Scientific) and filled with PBS, and a reference image was acquired. The RI-matching solution was added into the dish right after the PBS in the dish was removed, and time-lapse images were acquired for 1–2 hr. The image was divided into small tiles measuring 50 μm x 50 μm. The changes in intensity with time for each tile was fitted to a one-phase association function (${\rm{f}}({\rm{x}})={y}_{0}+(p-{y}_{0})\cdot [1-exp(-x/\tau )]$)^6^ to get the maximum values of transparency (P; plateau) and time constant (τ) of diffusion.

### Traumatic brain injury (TBI)

To induce cryogenic TBI, a metal probe with a diameter of 5 mm was cooled in liquid nitrogen. The head skin of anesthetized mice was cut, and the pre-chilled metal probe was placed in contact with the cranium of the mice for 30 seconds, as described previously^38^. The cut skin was sutured, and the animals were placed on a 37 °C hot plate until they were awake to be allowed to move freely in their home cage. Animals were sacrificed at 3, 7, and 14 days after TBI, and the whole brains were dissected and cleared by 6 hr ETC and were cut into the TBI region including the penumbra and undamaged periphery.

### Pulmonary fibrosis induced by ionizing radiation

As described previously^39^, 3-mm collimator was used for introducing the 90 Gy radiation damage to the left lung of C57BL/6 male mice (8 weeks). Radiation was delivered with an X-RAD 320 (Precision, North Branford, CT, USA), equipped with a collimator system with 5-cm-thick copper for focal radiation beams. Prior to irradiation, the mice were anesthetized with an intraperitoneally administered mixture of 30 mg/kg of zoletil and 10 mg/kg of rompun. The animals were scarified 4 weeks after irradiation. For lung harvesting, the mice were anesthetized with an intraperitoneally administered mixture of 30 mg/kg of Zoletil (tiletamine 25 mg/kg with zolazepam 25 mg/kg) and 10 mg/kg of Rompun (xylazine 10 mg/kg). Then 4% paraformaldehyde (PFA) solution was injected through trachea to fix the lung.

## Electronic supplementary material

Supplementary information

Supplementary Video 1
