# Supplementary material for: Optimizing tissue-clearing conditions based on analysis of the critical factors affecting tissue-clearing procedures
Source: Sci Rep. 2018 Aug 24;8:12815. doi: 10.1038/s41598-018-31153-7 (PMC6109102; doi:10.1038/s41598-018-31153-7)
Supplement: Supplementary file 1 — Supplementary information [file 41598_2018_31153_MOESM1_ESM.pdf]

Supplementary Information

Optimizing tissue-clearing conditions based on analysis of the critical factors affecting tissue-clearing procedures

June Hoan Kim[1], Min Jee Jang[1], Jungyoon Choi[1], Eunsoo Lee[1], Kyung–Deok Song [2,3], Jaeho Cho[4], Keun-Tae Kim[5], Hyuk-Jin Cha[6], Woong Sun[1*]

[1]Department of Anatomy, Korea University College of Medicine, Seoul 02841, Republic of Korea. [2]Department of Physics, Korea University, Seoul 02841, Republic of Korea. [3]Center for Molecular Spectroscopy and Dynamics, Institute for Basic Science (IBS), Seoul 02841, Republic of Korea. [4]Department of Radiation Oncology, Yonsei University College of Medicine, 50 Yonsei-ro, Seodaemun-gu, Seoul 120-752, Republic of Korea. [5]Department of Life Science, Sogang University, 35th Baekbum-ro Mapo-gu, Seoul 04107, Republic of Korea. [6]College of Pharmacy, Department of Pharmacy, Seoul National University, Gwanak-ro, Gwanak-gu, Seoul 08826, Republic of Korea.

[*]To whom correspondence should be addressed. Email: woongsun@korea.ac.kr

Supplementary information

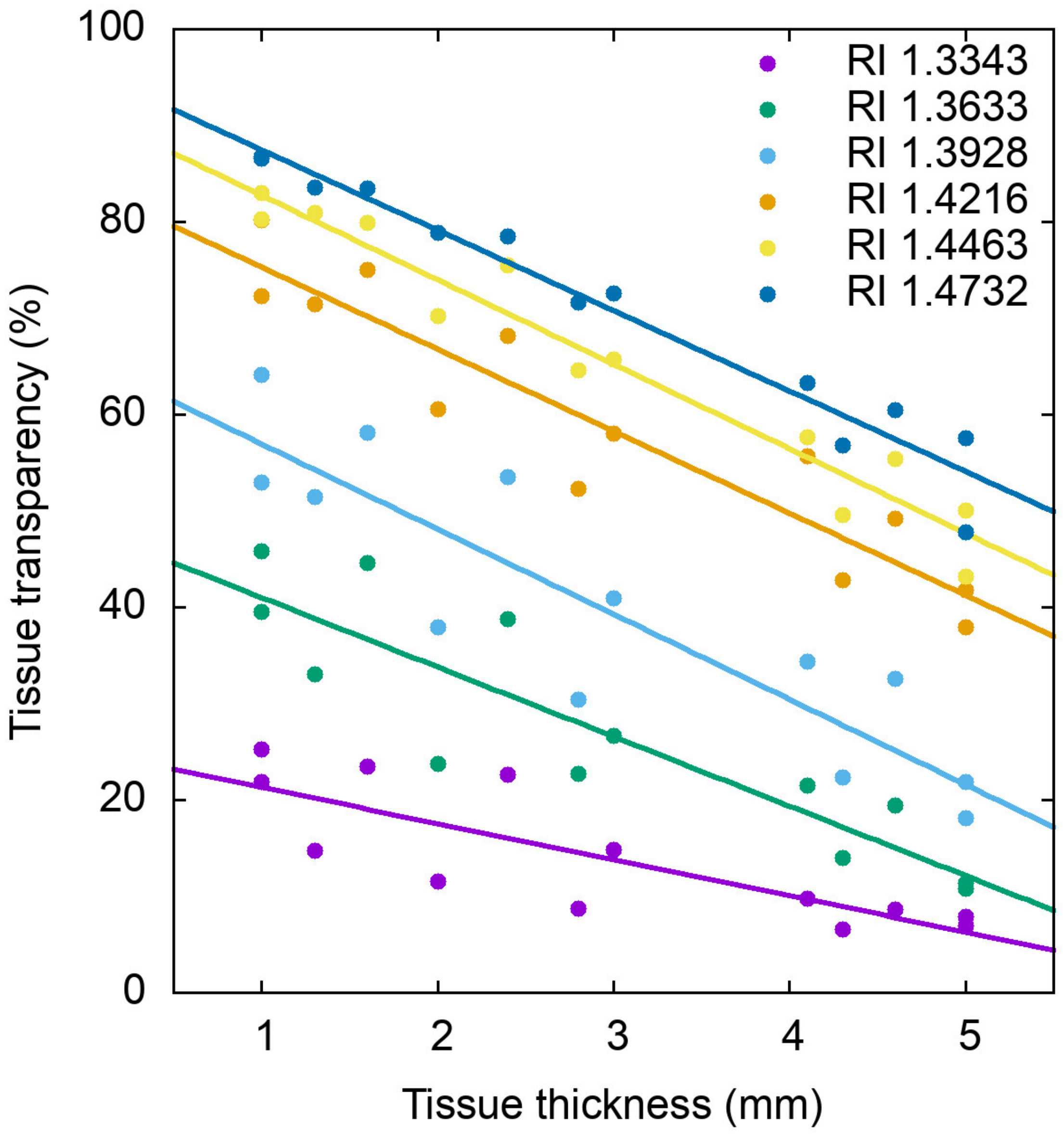

Supplementary Figure 1 Thickness and transparency of tissue. Whole liver tissues were cleared by ACT and was trimmed 1-5 mm-thick and RI-adjusted with different RI-matching solution; Cubic-mount was diluted by PBS to 0, 20, 40, 60, 80, 100 %, and transparency of the tissues measured. (N=13)

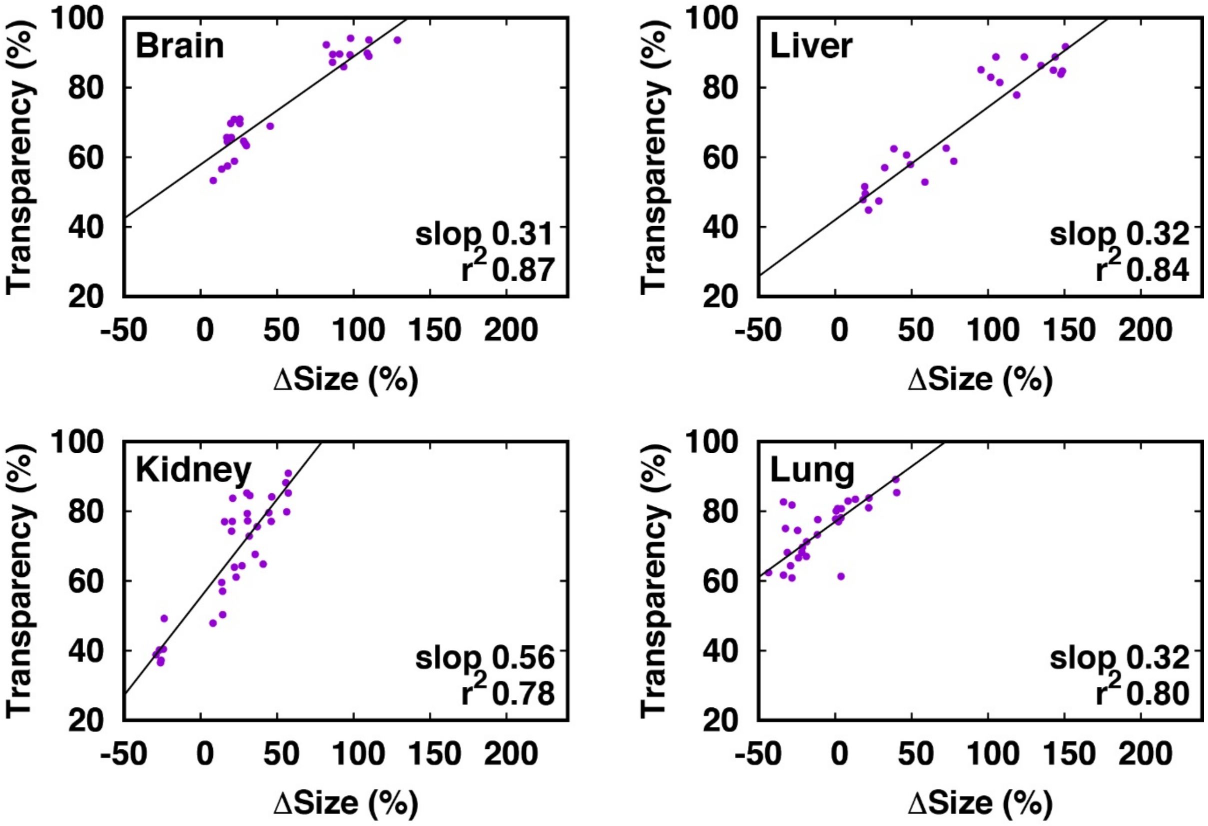

Supplementary Figure 2 Transparency change by size variation. Size change and transparency of tissues in 1-10x PBS and 4% SDS diluted in DI water were plotted and fitted by linear function for brain, liver, kidney, and lung tissues. (N=26, 24, 32, 30 for brain, liver, kidney and lung respectively)

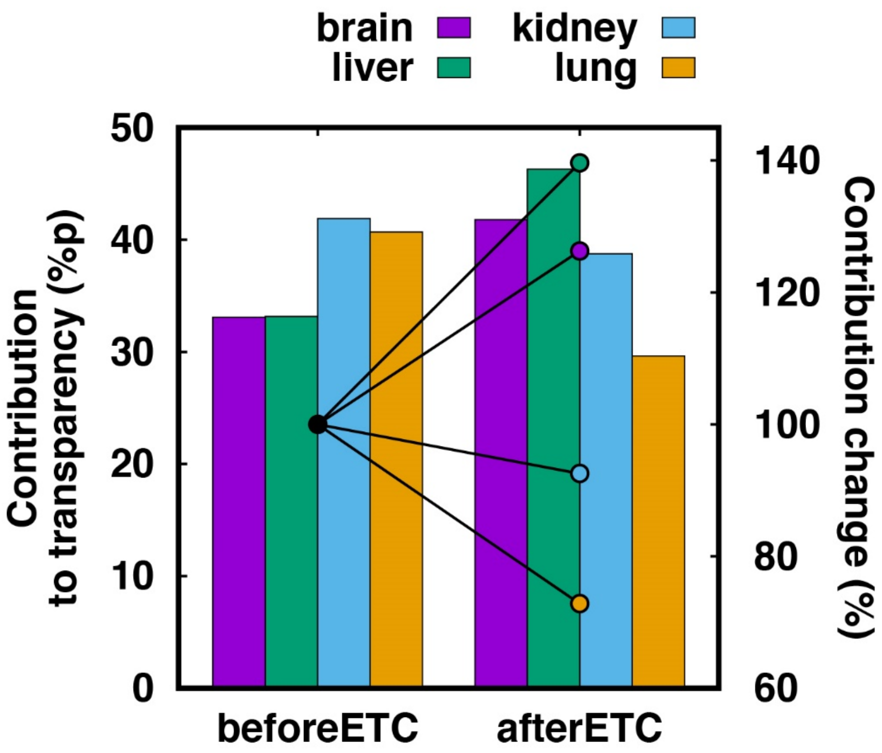

Supplementary Figure 3 The RI-matching of lipid and RI solution. Contributions of lipid to RI-matching before/after clearing (bars) and contribution changes by lipid extraction (lines). (N=3 for each organ).

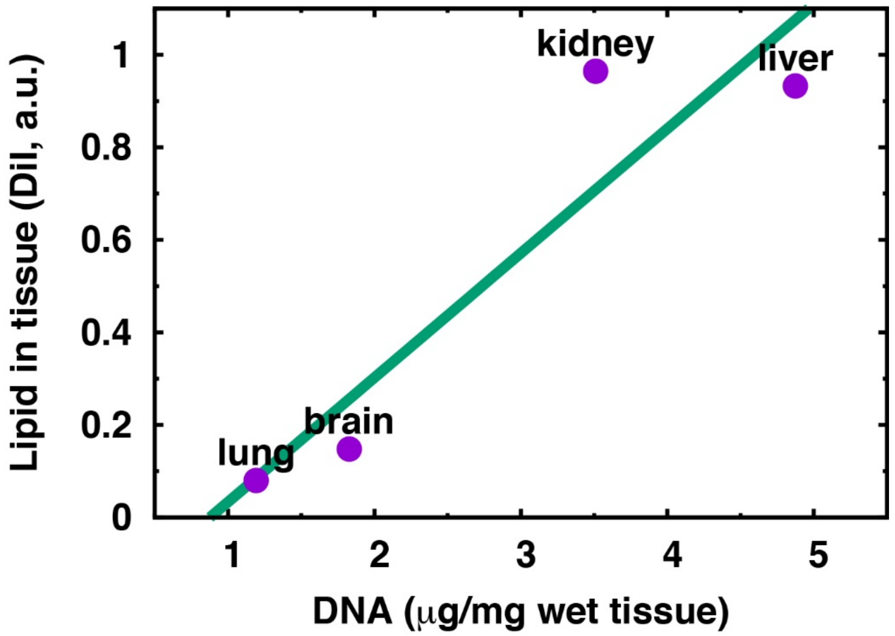

Supplementary Figure 4 DNA and lipid concentrations in tissues. Amount of lipid and DNA were average of 3 samples each for each organ. Samples for measuring lipid and DNA were different from each other.

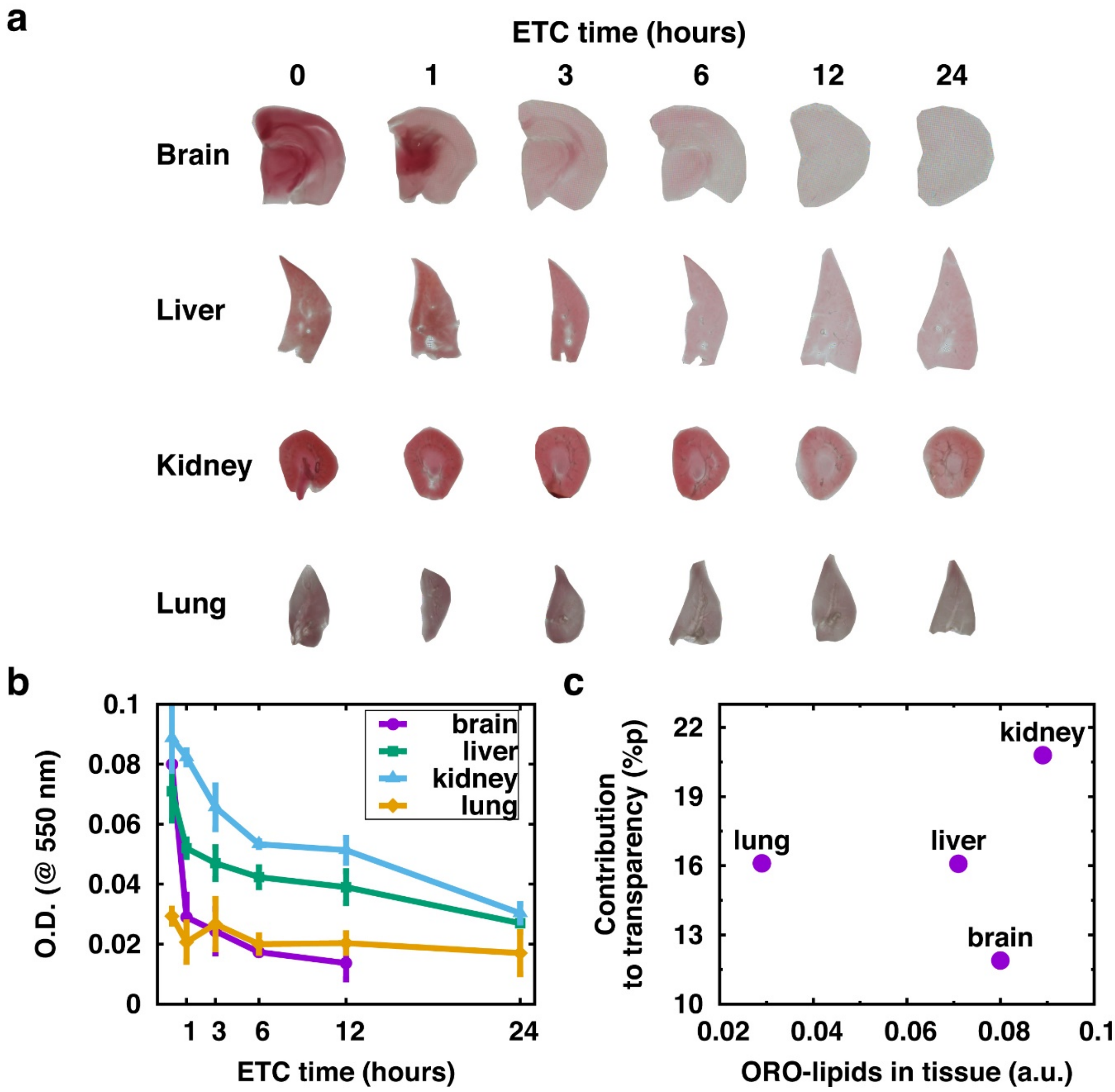

Supplementary Figure 5 Oil-red-O labeled lipid extraction. (A) Images of Oil-red-O loaded tissue, 1 mm-thick organ slices at different electrophoretic tissue clearing (ETC) time points. (B) Lipid extraction rate. (C) Correlation graph of extracted lipid and contribution of lipid extraction to improvement of transparency of each organ. (N=3 for each ETC time for each organ)

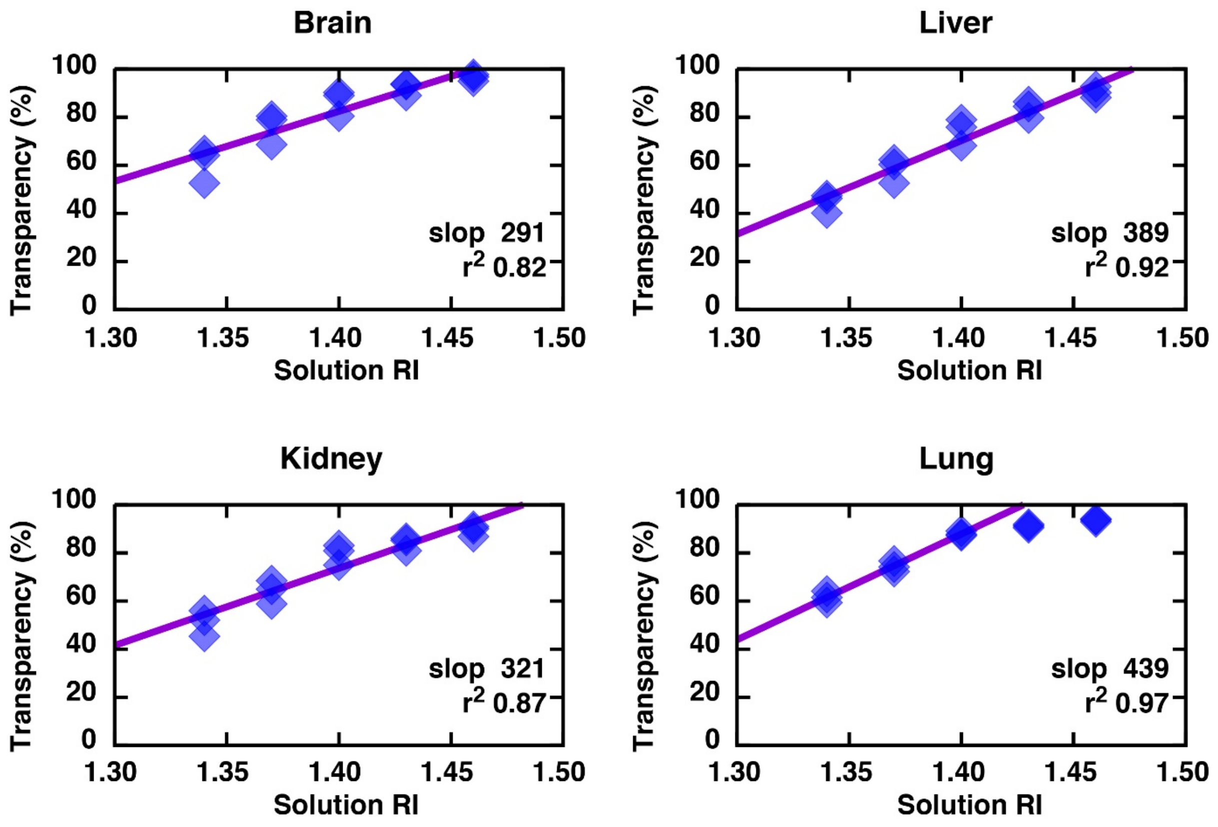

Supplementary Figure 6 The transparency variation by solution RI changes. Correlation of tissue transparency and RI value of solution. The data points were fitted by linear function. 4 points of lung which seemed to be saturated in transparency were excluded from linear regression. (N=3 for each organ).

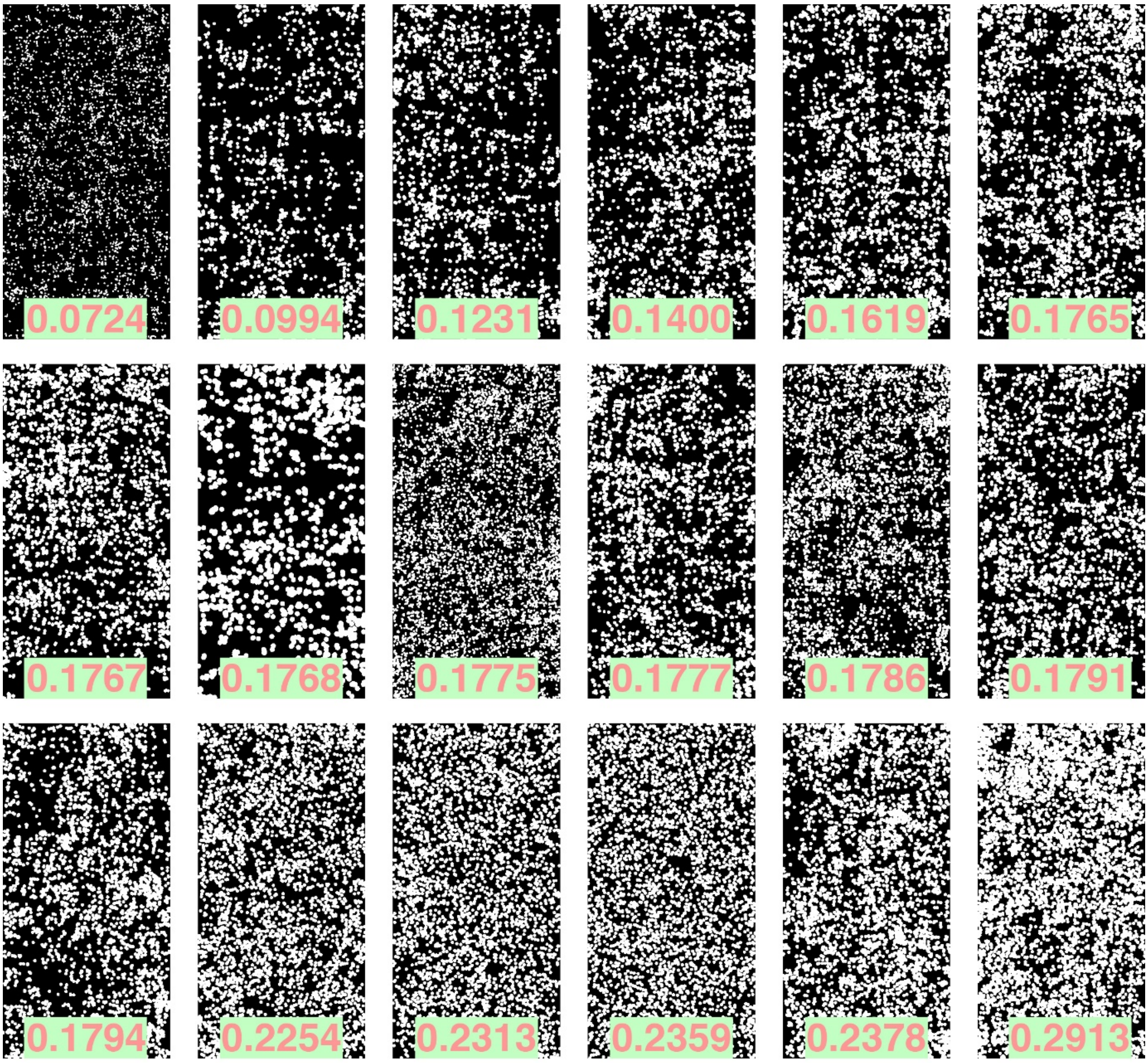

Supplementary Figure 7 Sample images with ECM density for FDTD simulation. Background (black) mimics background solution (water, RI: 1.33) and white dot represents ECM (RI: 1.47).

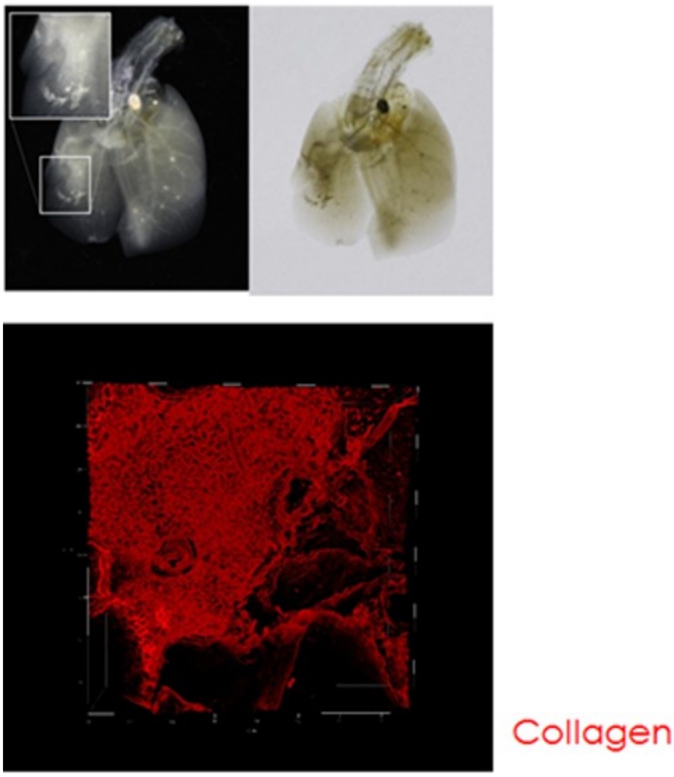

Supplementary Figure 8 The focal lung fibrosis model. The optical contrast of fibrosis that could be detected by collagen IV staining was enhanced by tissue clearing.

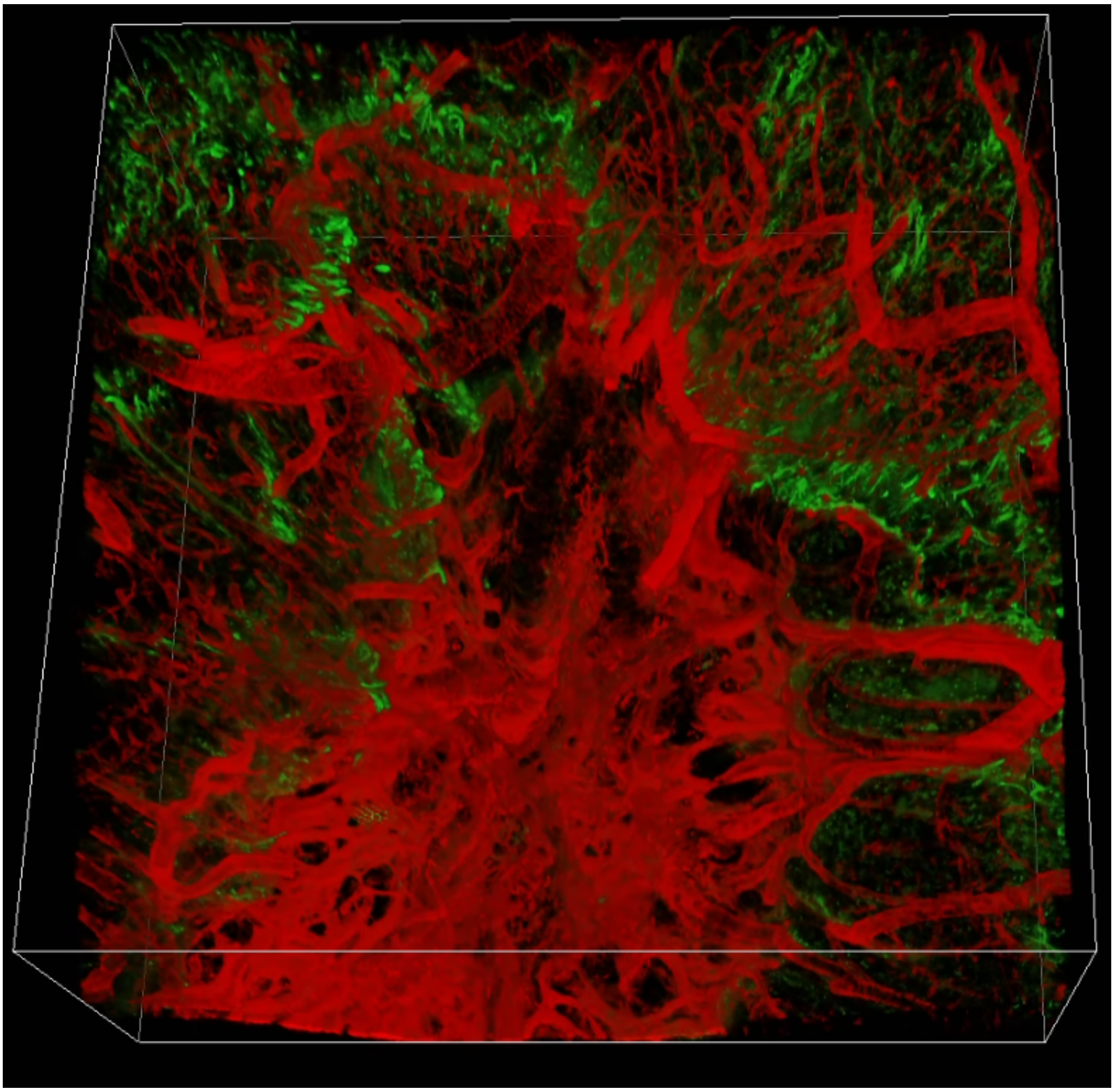

Supplementary Video 1 Accumulation of ECM and glial cells in and around TBI

A brain tissue in TBI days 7 was cut into 1.5 mm * 1.5 mm * 0.76 mm after 6hr ETC, and Collagen IV and GFAP were immunostained with red and green fluorescence respectively.

Supplementary Table 1. One-way ANOVA with post-hoc Tukey

* P < 0.05, ** P < 0.01, *** P < 0.001

Transparency					
	Tukey's Multiple Comparison Test	Mean Diff.	q	Summary	95% CI of diff
Brain	PBS(Fix-poly) vs CM(Fix-poly)	-33.08	21.56	***	-40.22 to -25.94
	PBS(Fix-poly) vs SDS(ETC)	-48.18	31.4	***	-55.32 to -41.04
	PBS(Fix-poly) vs PBS(ETC)	-11.89	7.748	**	-19.03 to -4.748
	PBS(Fix-poly) vs CM(ETC)	-53.68	34.98	***	-60.82 to -46.54
	CM(Fix-poly) vs SDS(ETC)	-15.1	9.84	***	-22.24 to -7.958
	CM(Fix-poly) vs PBS(ETC)	21.19	13.81	***	14.05 to 28.33
	CM(Fix-poly) vs CM(ETC)	-20.6	13.42	***	-27.74 to -13.46
	SDS(ETC) vs PBS(ETC)	36.29	23.65	***	29.15 to 43.43
	SDS(ETC) vs CM(ETC)	-5.5	3.584	ns	-12.64 to 1.642
	PBS(ETC) vs CM(ETC)	-41.79	27.23	***	-48.93 to -34.65
liver	PBS(Fix-poly) vs CM(Fix-poly)	-33.15	11.7	***	-46.34 to -19.96
	PBS(Fix-poly) vs SDS(ETC)	-41.75	14.73	***	-54.94 to -28.56
	PBS(Fix-poly) vs PBS(ETC)	-16.09	5.677	*	-29.28 to -2.898
	PBS(Fix-poly) vs CM(ETC)	-62.37	22	***	-75.56 to -49.18
	CM(Fix-poly) vs SDS(ETC)	-8.6	3.034	ns	-21.79 to 4.592
	CM(Fix-poly) vs PBS(ETC)	17.06	6.019	*	3.868 to 30.25
	CM(Fix-poly) vs CM(ETC)	-29.22	10.31	***	-42.41 to -16.03
	SDS(ETC) vs PBS(ETC)	25.66	9.053	***	12.47 to 38.85
	SDS(ETC) vs CM(ETC)	-20.62	7.275	**	-33.81 to -7.428
	PBS(ETC) vs CM(ETC)	-46.28	16.33	***	-59.47 to -33.09
kidney	PBS(Fix-poly) vs CM(Fix-poly)	-41.88	26.55	***	-49.22 to -34.54
	PBS(Fix-poly) vs SDS(ETC)	-58.06	36.8	***	-65.40 to -50.72
	PBS(Fix-poly) vs PBS(ETC)	-20.79	13.18	***	-28.13 to -13.45
	PBS(Fix-poly) vs CM(ETC)	-59.55	37.75	***	-66.89 to -52.21
	CM(Fix-poly) vs SDS(ETC)	-16.18	10.26	***	-23.52 to -8.838
	CM(Fix-poly) vs PBS(ETC)	21.09	13.37	***	13.75 to 28.43
	CM(Fix-poly) vs CM(ETC)	-17.67	11.2	***	-25.01 to -10.33
	SDS(ETC) vs PBS(ETC)	37.27	23.62	***	29.93 to 44.61
	SDS(ETC) vs CM(ETC)	-1.49	0.9445	ns	-8.832 to 5.852
	PBS(ETC) vs CM(ETC)	-38.76	24.57	***	-46.10 to -31.42
lung	PBS(Fix-poly) vs CM(Fix-poly)	-40.69	16.49	***	-52.17 to -29.21
	PBS(Fix-poly) vs SDS(ETC)	-31.26	12.67	***	-42.74 to -19.78
	PBS(Fix-poly) vs PBS(ETC)	-16.14	6.542	**	-27.62 to -4.658
	PBS(Fix-poly) vs CM(ETC)	-47	19.05	***	-58.48 to -35.52

	CM(Fix-poly) vs SDS(ETC)	9.43	3.822	ns	-2.052 to 20.91
	CM(Fix-poly) vs PBS(ETC)	24.55	9.951	***	13.07 to 36.03
	CM(Fix-poly) vs CM(ETC)	-6.31	2.558	ns	-17.79 to 5.172
	SDS(ETC) vs PBS(ETC)	15.12	6.129	*	3.638 to 26.60
	SDS(ETC) vs CM(ETC)	-15.74	6.38	**	-27.22 to -4.258
	PBS(ETC) vs CM(ETC)	-30.86	12.51	***	-42.34 to -19.38
**Size**					
	PBS(Fix-poly) vs CM(Fix-poly)	35.33	4.984	*	2.341 to 68.32
Brain	PBS(Fix-poly) vs SDS(ETC)	-93.72	13.22	***	-126.7 to -60.73
	PBS(Fix-poly) vs PBS(ETC)	-34.82	4.912	*	-67.81 to -1.831
	PBS(Fix-poly) vs CM(ETC)	7.66	1.081	ns	-25.33 to 40.65
	CM(Fix-poly) vs SDS(ETC)	-129.1	18.21	***	-162.0 to -96.06
	CM(Fix-poly) vs PBS(ETC)	-70.15	9.897	***	-103.1 to -37.16
	CM(Fix-poly) vs CM(ETC)	-27.67	3.904	ns	-60.66 to 5.319
	SDS(ETC) vs PBS(ETC)	58.9	8.31	**	25.91 to 91.89
	SDS(ETC) vs CM(ETC)	101.4	14.3	***	68.39 to 134.4
	PBS(ETC) vs CM(ETC)	42.48	5.993	*	9.491 to 75.47
	PBS(Fix-poly) vs CM(Fix-poly)	-20.75	9.202	***	-31.24 to -10.26
liver	PBS(Fix-poly) vs SDS(ETC)	-143.6	63.67	***	-154.1 to -133.1
	PBS(Fix-poly) vs PBS(ETC)	-130.2	57.76	***	-140.7 to -119.8
	PBS(Fix-poly) vs CM(ETC)	-42.15	18.69	***	-52.64 to -31.66
	CM(Fix-poly) vs SDS(ETC)	-122.8	54.47	***	-133.3 to -112.3
	CM(Fix-poly) vs PBS(ETC)	-109.5	48.56	***	-120.0 to -99.00
	CM(Fix-poly) vs CM(ETC)	-21.4	9.491	***	-31.90 to -10.91
	SDS(ETC) vs PBS(ETC)	13.32	5.909	*	2.830 to 23.82
	SDS(ETC) vs CM(ETC)	101.4	44.98	***	90.93 to 111.9
	PBS(ETC) vs CM(ETC)	88.1	39.07	***	77.60 to 98.59
	PBS(Fix-poly) vs CM(Fix-poly)	-1.849	0.7198	ns	-13.81 to 10.11
kidney	PBS(Fix-poly) vs SDS(ETC)	-49.39	19.22	***	-61.35 to -37.43
	PBS(Fix-poly) vs PBS(ETC)	-21.02	8.181	**	-32.97 to -9.061
	PBS(Fix-poly) vs CM(ETC)	2.833	1.103	ns	-9.124 to 14.79
	CM(Fix-poly) vs SDS(ETC)	-47.54	18.5	***	-59.50 to -35.58
	CM(Fix-poly) vs PBS(ETC)	-19.17	7.461	**	-31.12 to -7.211
	CM(Fix-poly) vs CM(ETC)	4.682	1.822	ns	-7.275 to 16.64
	SDS(ETC) vs PBS(ETC)	28.37	11.04	***	16.41 to 40.33
	SDS(ETC) vs CM(ETC)	52.22	20.33	***	40.26 to 64.18
	PBS(ETC) vs CM(ETC)	23.85	9.283	***	11.89 to 35.81
	PBS(Fix-poly) vs CM(Fix-poly)	-19.48	7.097	**	-32.26 to -6.705
lung	PBS(Fix-poly) vs SDS(ETC)	-4.533	1.651	ns	-17.31 to 8.243
	PBS(Fix-poly) vs PBS(ETC)	27.32	9.951	***	14.54 to 40.09

	PBS(Fix-poly) vs CM(ETC)	15.84	5.77	*	3.063 to 28.61
	CM(Fix-poly) vs SDS(ETC)	14.95	5.445	*	2.172 to 27.72
	CM(Fix-poly) vs PBS(ETC)	46.8	17.05	***	34.02 to 59.57
	CM(Fix-poly) vs CM(ETC)	35.32	12.87	***	22.54 to 48.09
	SDS(ETC) vs PBS(ETC)	31.85	11.6	***	19.07 to 44.62
	SDS(ETC) vs CM(ETC)	20.37	7.421	**	7.596 to 33.15
	PBS(ETC) vs CM(ETC)	-11.48	4.181	ns	-24.25 to 1.298
	PBS(ETC) vs CM(ETC)	-11.48	4.181	ns	-24.25 to 1.298
